# When Time Precludes Certainty: Intravenous Tenecteplase for Acute Global Aphasia in a Young Woman Along With Post-acute Diagnostic Reorientation

**DOI:** 10.7759/cureus.109338

**Published:** 2026-05-21

**Authors:** Adniel García Cruz, Jhonatan A Rojas Cristancho, Gretel Pimentel Barreiro, Alicia M Ramirez Mendez, Alena Arbos Aguilar

**Affiliations:** 1 Geriatrics, Hospital Universitari Arnau de Vilanova Lleida, Lleida, ESP; 2 Neurology, Hospital Universitari Arnau de Vilanova Lleida, Lleida, ESP; 3 Family Medicine, Hospital Universitari Arnau de Vilanova Lleida, Lleida, ESP

**Keywords:** acute aphasia, diagnostic uncertainty, handl syndrome, intravenous thrombolysis, migraine with aura, stroke code, stroke mimic, tenecteplase, therapeutic window, young adult

## Abstract

Stroke mimics represent a major diagnostic challenge in the hyperacute setting. We report the case of a 29-year-old woman with no prior documented history of migraine or clinically significant headache who developed sudden-onset bradylalia and right hemisensory paresthesias, rapidly progressing to severe mixed aphasia with motor predominance, jargonaphasia, anomia, dysgraphia, alexia, and involuntary mixing of three languages. The initial National Institutes of Health Stroke Scale (NIHSS) score was five, accompanied by headache and vomiting. Intense agitation required procedural sedation for CT acquisition. Motion artifact rendered perfusion maps non-interpretable. Non-contrast CT showed no hemorrhage or established infarct (Alberta stroke programme early CT score (ASPECTS) 10), and CT angiography demonstrated no large vessel occlusion. Given the disabling neurological deficit and symptom onset within the therapeutic window, intravenous tenecteplase 17.5 mg was administered. Neurological recovery was complete within hours, and the delayed brain MRI was normal. Headache with Neurological Deficits and Cerebrospinal Fluid Lymphocytosis (HaNDL) syndrome remained an unconfirmed diagnostic consideration. Ultimately, the episode was assessed as a probable stroke mimic, most consistent with migraine with aphasic aura. This case illustrates that intravenous thrombolysis may be clinically justifiable under reasonable diagnostic uncertainty when the deficit is severe, the therapeutic window is favorable, and imaging is technically limited, followed by cautious post-acute diagnostic reorientation.

## Introduction

Distinguishing acute ischemic stroke from its mimics in the hyperacute setting remains a demanding challenge in emergency neurology. Stroke mimics are frequent among patients activated under a stroke code, and acute aphasia can arise from both ischemic stroke and transient mimicking etiologies [1-8]. In the presence of a disabling neurological deficit, treatment decisions within the therapeutic window must often rely on initial severity, time from onset, and available emergent neuroimaging, rather than absolute diagnostic certainty. Available evidence suggests that intravenous thrombolysis in stroke mimics is associated with low rates of symptomatic intracranial hemorrhage [9-11]. This case illustrates a clinically defensible decision for acute thrombolysis followed by cautious post-acute diagnostic reorientation.

## Case presentation

Background and baseline status

A 29-year-old woman with no known drug allergies was evaluated in the emergency department. Relevant medical history included hypothyroidism managed with levothyroxine 125 mcg/day. She was overweight, and this was documented in prior medical records based on available previous anthropometric data (BMI 26.5 kg/m²). She had a prior pregnancy complicated by preeclampsia, which resolved without documented residual sequelae. At baseline, she was fully independent, professionally active, and had a modified Rankin Scale (mRS) score of 0 [12]. No personal or family history of migraine or clinically significant headache was documented.

Symptom onset and clinical progression

On day one, at 20:00 hours, the patient developed sudden-onset bradylalia and right hemisensory paresthesias. Over the following minutes and hours, the deficit progressed to a predominantly motor mixed aphasia with jargonaphasia, semantic and phonemic paraphasias, severe anomia, dysgraphia, alexia, and involuntary mixing of three languages (Catalan, Spanish, and English). Family members reported headache as an accompanying symptom from the onset, and she experienced a single episode of vomiting.

Emergency department evaluation and neurological examination

The patient arrived at the emergency department at 21:54 hours on day one and was initially triaged for nausea and vomiting. Her progressive language deterioration was subsequently recognized by the nursing staff during her ED stay, prompting urgent neurology consultation, and a stroke code was activated at 22:52 hours. On examination, she demonstrated profound aphasia, repetitive speech output, partial compliance with simple commands, imitative gestural behavior, and bilateral Hoffmann signs. The National Institutes of Health Stroke Scale (NIHSS) score was five [13]. Vital signs at neurological evaluation were blood pressure 110/69 mmHg, heart rate 90 bpm, oxygen saturation 96% on room air, and afebrile status.

Emergent neuroimaging and technical limitations

**Figure 1:**
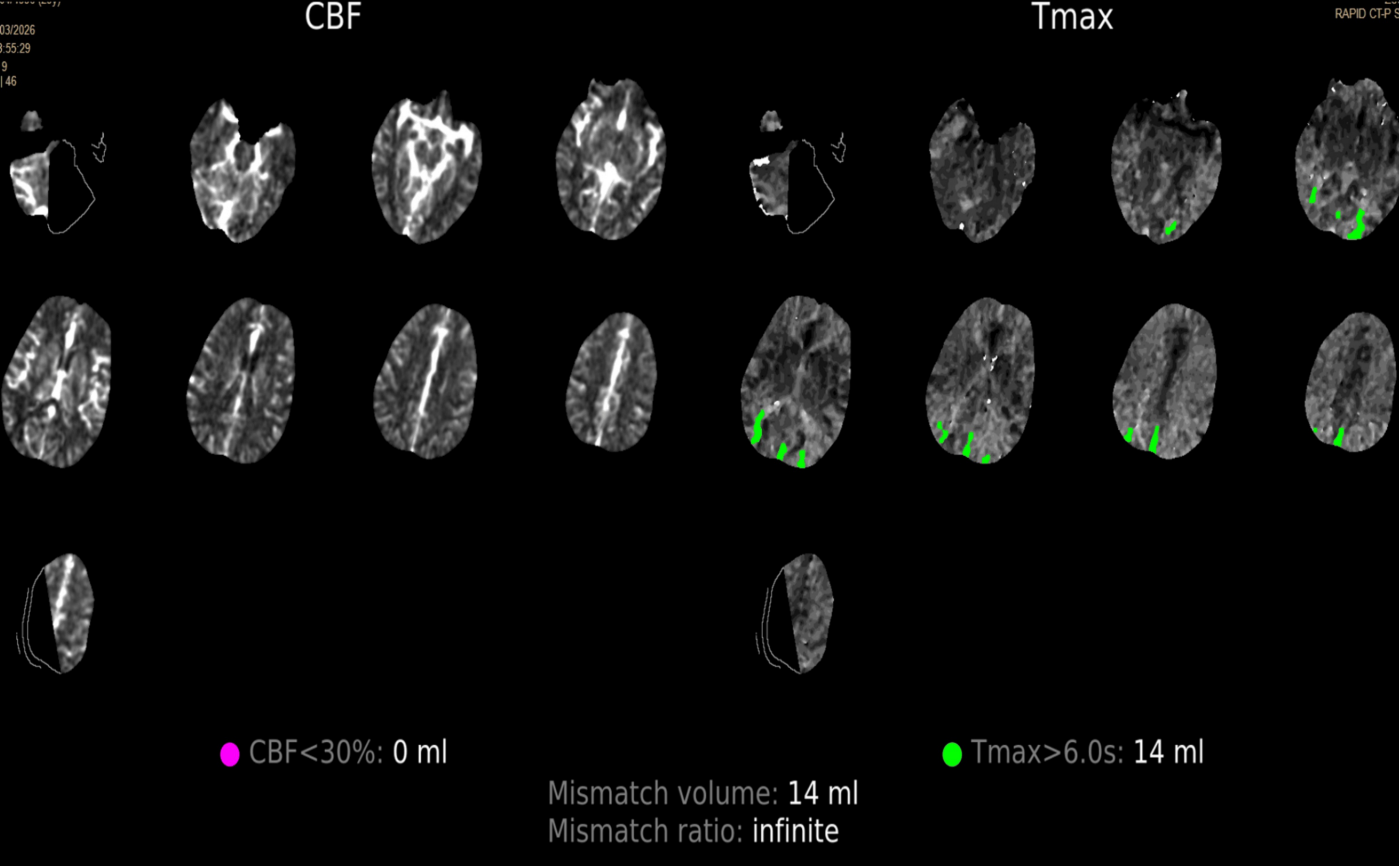
CT perfusion maps from the hyperacute stroke code study A non-specific apparent perfusion defect is visible and was reported by radiology as an artifact; the green Tmax signal corresponds to the motion-related artifact.

Intense patient agitation, attributable to aphasia-induced disorientation, precluded adequate multimodal CT acquisition without pharmacological sedation. Based on the available medical history, she was considered an estimated American Society of Anesthesiologists physical status II and underwent monitored procedural sedation in the CT suite with midazolam 4 mg IV followed by sequential propofol doses (60 mg + 40 mg). No procedural complications were documented. Persistent motion artifacts required the multimodal CT protocol to be repeated three times, and perfusion maps were non-interpretable (Figure 1).

**Figure 2:**
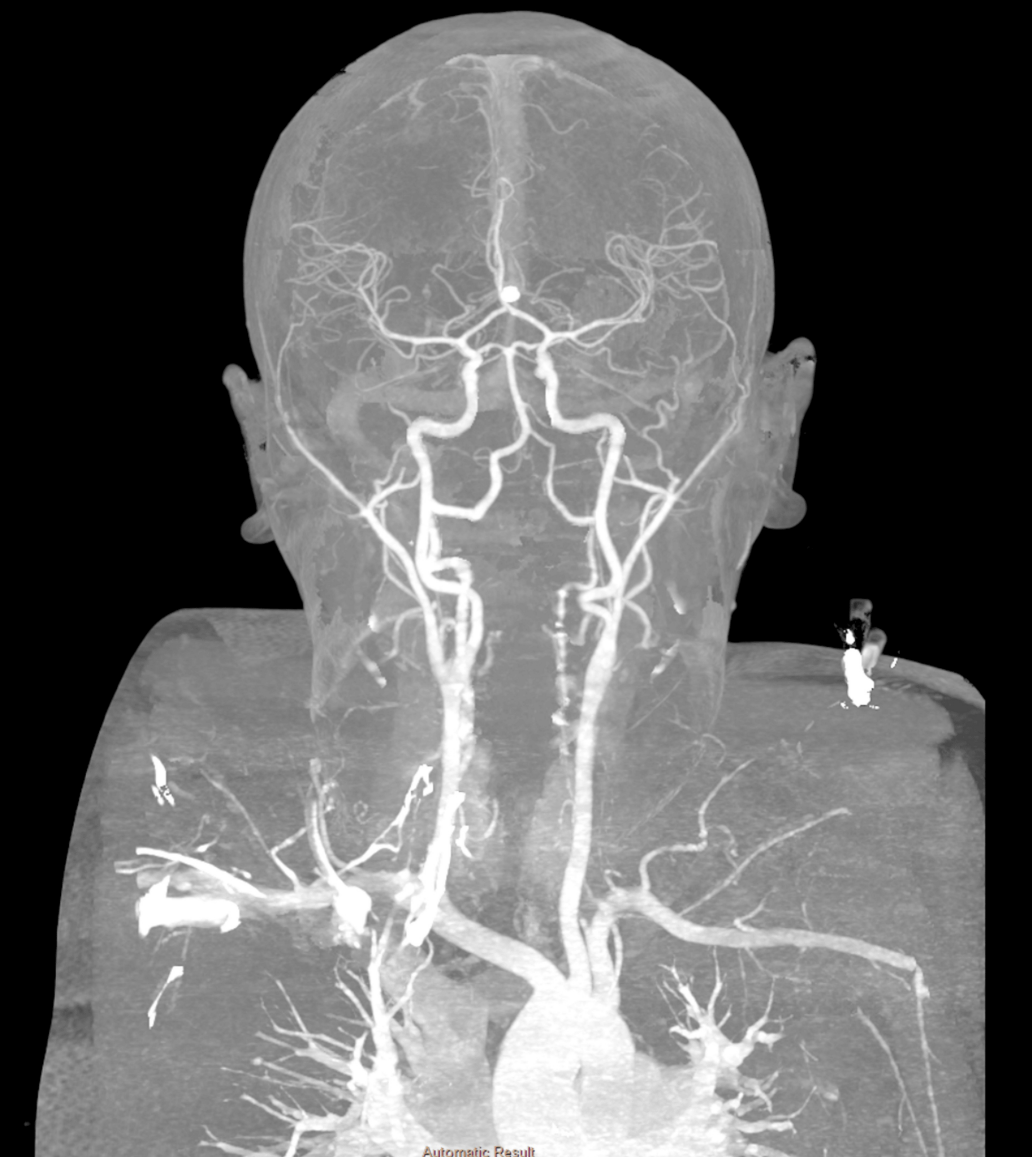
CT angiography reconstruction from the stroke code protocol showing no large vessel occlusion

Non-contrast cranial CT showed no acute hemorrhagic lesions and no features suggestive of established hyperacute infarction (Alberta stroke programme early CT score (ASPECTS) 10). CT angiography demonstrated patency of the major cerebral arteries (bilateral anterior cerebral artery (ACA), middle cerebral artery (MCA), and posterior cerebral artery (PCA)) and the vertebrobasilar system, with no large vessel occlusion or significant stenosis (Figure 2).

Clinical reasoning and therapeutic decision

The clinical presentation raised a reasonable suspicion of acute ischemic stroke. Given the clinical severity, withholding treatment could have resulted in persistent neurological disability if the episode represented an ischemic stroke. The family was informed of the diagnostic uncertainty, potential benefits, and risks of thrombolysis. In the absence of absolute or relative contraindications, IV tenecteplase 17.5 mg was administered as a bolus at 00:14 hours on day two, with a door-to-needle time of two hours and 20 minutes.

In-hospital evolution

Neurological recovery was progressive and complete. On reassessment on the morning of day two, the patient was fully oriented with normalized speech (NIHSS 0). She reported a migrainous headache that gradually resolved with conventional analgesia. Hemodynamic monitoring showed a trend toward asymptomatic arterial hypotension; available documented blood pressure values remained within a similar low-normal range, including 106/66 mmHg on day three, without symptomatic hypotension or clinically significant hemodynamic instability.

**Figure 3:**
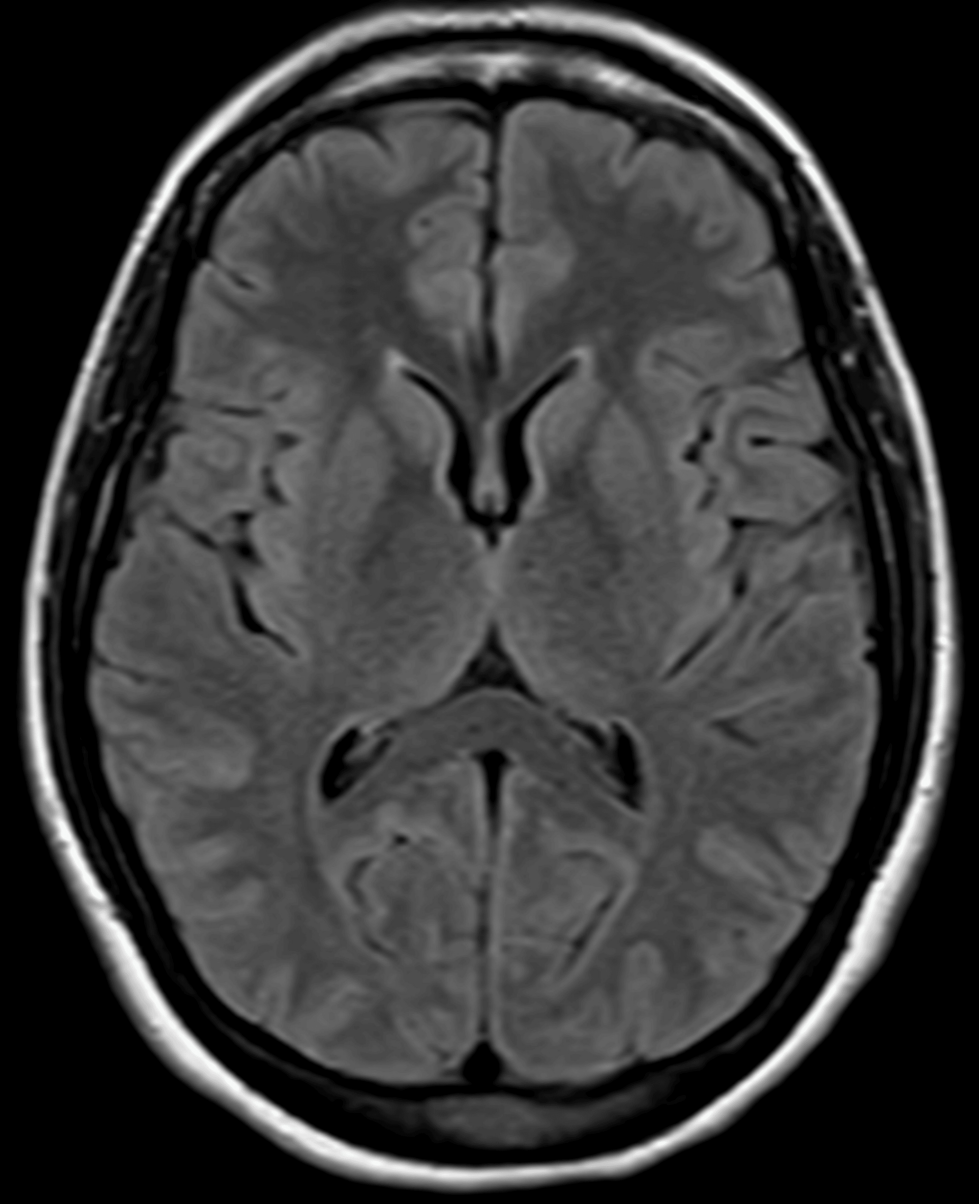
Axial brain MRI, FLAIR sequence, reported as within normal limits FLAIR: Fluid-Attenuated Inversion Recovery.

**Figure 4:**
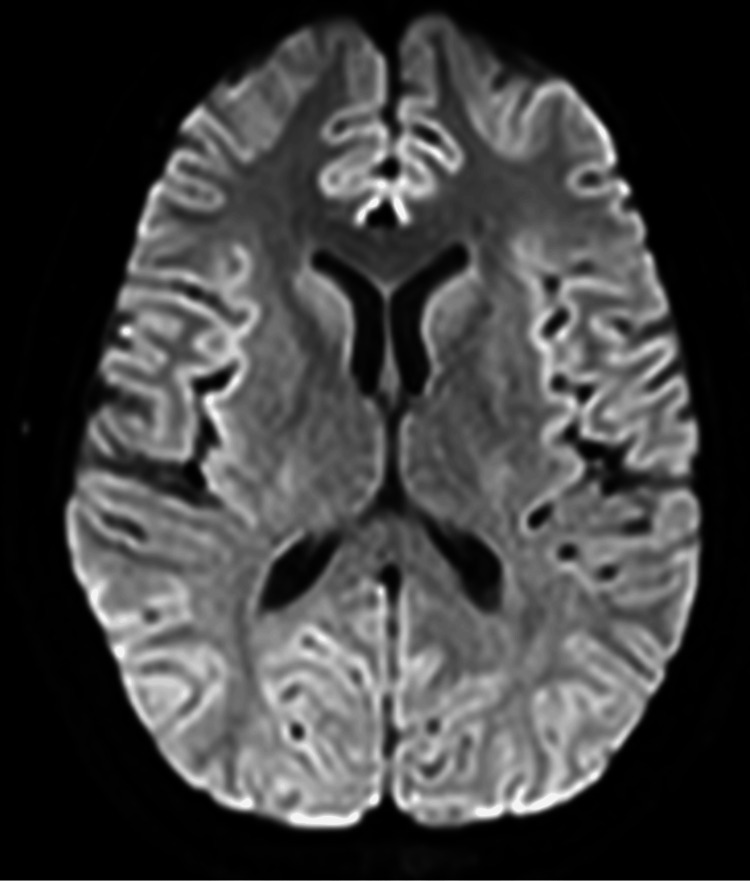
Axial brain MRI, diffusion-weighted imaging sequence, without diffusion restriction

Brain Positron Emission Tomography/Computed Tomography (PET-CT) on day three showed no detectable cerebral metabolic abnormalities; however, this finding had limited discriminatory value and did not influence the initial thrombolysis decision. Headache with Neurological Deficits and Cerebrospinal Fluid Lymphocytosis (HaNDL) syndrome was considered during admission but remained unconfirmed. Lumbar puncture was planned for post-acute evaluation but was ultimately declined by the patient after neurological recovery; therefore, cerebrospinal fluid (CSF) analysis was not available. Brain MRI without contrast on day six was normal, showing no diffusion restriction on diffusion-weighted imaging (DWI) sequences (Figures 3, 4).

The patient was discharged on day six.

Outpatient follow-up

At primary care follow-up on day 15, the clinical episode was assessed as a probable stroke mimic, most consistent with migraine with aphasic aura. The patient reported subsequent similar episodes with good response to naproxen. This interpretation remains retrospective and probabilistic rather than definitive. The clinical timeline is summarized in Table 1.

**Table 1:** Clinical episode timeline NIHSS: National Institutes of Health Stroke Scale.

Day (time)	Event
Day 1 (20:00)	Sudden onset of bradylalia and right hemisensory paresthesias, progressing to severe aphasia
Day 1 (21:54)	Arrival at the emergency department
Day 1 (22:52)	Stroke code activation
Day 2 (00:14)	IV tenecteplase 17.5 mg administered
Day 2 (10:03)	Neurological reassessment: NIHSS 0; migrainous headache
Day 6 (09:41)	Brain MRI without contrast: normal study, without diffusion restriction
Day 15	Primary care follow-up: considered most consistent with migraine with aura

The main diagnostic considerations during admission and follow-up are summarized in Table 2.

**Table 2:** Differential diagnosis summary TIA: Transient ischemic attack; NIHSS: National Institutes of Health Stroke Scale; HanDL: Headache and Neurologic Deficits with cerebrospinal fluid Lymphocytosis.

Diagnosis	Assessment in this case
Acute ischemic stroke/TIA	Plausible hyperacute hypothesis given sudden disabling aphasia, NIHSS 5, right hemisensory symptoms, and therapeutic window; completed infarction was not confirmed by delayed MRI.
Migraine with aphasic aura	Most likely post-acute interpretation based on clinical evolution, normal MRI, outpatient assessment, and recurrent similar episodes responsive to naproxen.
HaNDL syndrome	Considered during admission but unconfirmed because CSF analysis was not obtained.
Postictal aphasia	Considered less likely; not entirely excluded because EEG was not available.
Cerebral venous thrombosis	Less likely based on available CT angiography data, but not completely excluded.

Ethical statement

This report was prepared in accordance with the CAse REports (CARE) guidelines [14]. The case was fully anonymized, and no directly or indirectly identifiable personal data was included. Patient confidentiality was maintained throughout. Given the retrospective and fully anonymized nature of this report, formal written informed consent was not required as per institutional policies.

## Discussion

This case illustrates the complex decision-making process required when severe acute aphasia presents in the emergency department with technically limited neuroimaging. First, the rationale for administering tenecteplase rested on a risk-benefit analysis under time-sensitive uncertainty. The patient exhibited a clearly disabling focal deficit (NIHSS 5) within the therapeutic window, and non-contrast CT excluded hemorrhage. The American Heart Association and the American Stroke Association (AHA/ASA) guidelines state that advanced imaging should not delay thrombolysis when clinical suspicion is high [15]. Because non-interpretable perfusion maps deprived the team of a potentially useful diagnostic tool, the decision prioritized treatment of a possible disabling ischemic stroke over absolute diagnostic certainty. Available evidence suggests a low rate of symptomatic intracranial hemorrhage among thrombolysis-treated stroke mimics [9-11].

Second, post-acute reevaluation highlighted the importance of cautious diagnostic reorientation. Complete neurological recovery to NIHSS 0 and the absence of diffusion restriction on the delayed brain MRI made established ischemic infarction unlikely. Consequently, the clinical picture favored a probable stroke mimic. Finally, while HaNDL syndrome was considered during admission, the absence of CSF analysis rendered this hypothesis unconfirmed. PET-CT was non-contributory and had limited discriminatory value in this context. Conversely, outpatient follow-up documented recurrent similar episodes with a favorable response to naproxen, supporting the outpatient assessment of migraine with aura. Therefore, the most scientifically prudent interpretation is a probable stroke mimic, most consistent with migraine with aphasic aura. This divergence underscores the value of structured outpatient follow-up in refining diagnoses that remain uncertain at hospital discharge.

## Conclusions

This case does not prove a final etiological diagnosis; rather, it illustrates a defensible hyperacute thrombolysis decision followed by cautious post-acute diagnostic reorientation. Sudden-onset severe global aphasia with technically limited emergent neuroimaging constituted a reasonable indication for intravenous tenecteplase, given the disabling deficit, therapeutic window, and exclusion of hemorrhage. Complete recovery, normal delayed MRI, and subsequent outpatient follow-up favored a probable stroke mimic, most consistent with migraine with aphasic aura, while HaNDL syndrome remained an unconfirmed possibility. Ultimately, thrombolysis may be clinically justified under reasonable diagnostic uncertainty, but final etiological conclusions must remain strictly proportional to the available objective evidence.
